# Pretreatment with Silver Thiosulfate Increases the Auxin-Inductive Effect for Rooting Mature Chestnut Shoots

**DOI:** 10.3390/plants14243756

**Published:** 2025-12-10

**Authors:** Ricardo Castro-Camba, Conchi Sánchez, Saleta Rico, Nieves Vidal, Anxela Aldrey, María José Cernadas, Purificación Covelo, Jesús M. Vielba

**Affiliations:** Department of Plant Production, Misión Biológica de Galicia (CSIC), Avda de Vigo s/n, 15705 Santiago de Compostela, Spain; ricardo.castro@csic.es (R.C.-C.); saleta@mbg.csic.es (S.R.); nieves@mbg.csic.es (N.V.);

**Keywords:** adventitious roots, auxin, chestnut, ethylene, mature shoots, recalcitrance, silver thiosulfate

## Abstract

Chestnut is a highly valuable species at both the ecological and economic levels, but vegetative propagation protocols have not been optimized for mature material due to its recalcitrant behaviour, thus limiting potential biotechnological applications. In this study, we focused on the formation of adventitious roots in mature chestnut microshoots, which show low rooting responses. Silver thiosulfate, an ethylene-signalling inhibitor, was applied as a pretreatment before auxin induction to study the role of ethylene in this developmental process. Rooting rate was significantly improved in response to the pretreatment, suggesting that ethylene negatively influences the induction of adventitious roots in mature shoots. Transcriptomics and real-time PCR analyses suggest that the improvement in the rooting response was mainly due to the activity of different auxin transport genes, whose expression seems to be repressed by ethylene. However, other hormones might also be negatively influencing rooting in mature shoots, although their specific role remains to be characterized.

## 1. Introduction

Natural forests and orchards of chestnut trees (*Castanea sativa* Mill.) are important ecosystems in many countries in the Mediterranean basin. These ecosystems provide a variety of services at socio-economic and ecological levels, such as nuts, timber, mushrooms, and recreational areas, as well as acting as biodiversity refuges and carbon sinks. Furthermore, when managed correctly, orchards can contribute to the prevention and mitigation of wildfires through biomass removal and landscape mosaic design. However, these populations are at risk due to the impact of climate change, which alters phenology, physiology, and reduces intra-species diversity [1], while the loss of traditional management practices might also have a negative impact [2]. Modelling analyses suggest that chestnut populations in Europe are threatened by climate change due to the expected increase in drought and extreme weather events [3,4], as well as a surge in the activity of common Pathogens (i.e., phytophthora spp.) [5,6]. Therefore, there is an urgent need to improve our knowledge of the available chestnut genetic pool in order to identify climate-resilient and pathogen-tolerant genotypes, as a strategy to alleviate the dangerous effects of these threats [5,7]. However, these genotypes must be asexually propagated to preserve their valuable traits.

Vegetative propagation is the most suitable method for multiplying selected genotypes. However, chestnut is a recalcitrant species that exhibits a low rate of positive morphogenetic responses, particularly at the mature stage. Genotype quality can only be assessed in adult material, and ageing is the main factor constraining the propagation of the selected varieties [8]. This limitation hinders the application of protocols, including in vitro micropropagation. Rooting protocols optimized for juvenile-like chestnut microshoots (auxin type and concentration, rooting media, etc.) have been proven ineffective for mature microshoots, which are difficult to root. Other factors, such as the genotype or the light regime, might also influence the success of the process, but low rooting rates are generally achieved for mature material [9]. The process of adventitious rooting (AR) is divided into four sequential steps: dedifferentiation, induction, initiation, and expression [10,11]. In the basal section of the shoots, specific cells within the vascular bundles or flanking tissues must shift their ongoing genetic programme and acquire a new developmental fate by extensively modifying their transcriptome [12,13]. Auxin is the triggering agent and the most relevant phytohormone regulating AR, and its uneven distribution between neighbouring tissues as a consequence of the activity of the auxin polar transport machinery serves as the critical developmental cue for the dedifferentiation and formation of root initial cells [14]. This auxin gradient specifies a predefined developmental transition that is dependent on the specific context of the cell [15]. However, auxin signalling occurs at multiple levels and is closely linked to the signalling pathways of other hormones (e.g., ethylene, gibberellins, and abscisic acid [16]), adding extra regulatory layers to the AR process. Moreover, wounding, which is necessary for AR, also induces changes in the levels of phytohormones, among other physiological responses [17,18].

The ability of juvenile-like chestnut microshoots to form adventitious roots (ARs) has been analyzed at physiological and transcriptomic levels. Optimized protocols have been developed to obtain healthy, rooted shoots [19]. However, the rooting of mature microshoots remains to be refined. Previous reports have suggested that the lack of rooting responses in mature shoots might be due to gene expression in response to specific hormones, such as ethylene (ET) [20]. It is known that the cross-talk between auxin and ET takes place at the signalling, transport, and metabolic levels [11]. The application of compounds that modify ET content or signalling has been shown to have a limited but significant effect on the ability of mature microshoots to form ARs [21]. In the present study, a modified protocol was employed, involving the pretreatment of shoots with silver thiosulfate (STS), a known inhibitor of ET signalling, prior to the application of exogenous auxin. The physiological results observed here were similar to those obtained when ET signalling or synthesis inhibitors were applied concomitantly with auxin [21]. A transcriptomics analysis was performed at specific time-point to determine the changes in gene expression triggered by STS pretreatment compared to auxin alone. Additionally, the expression of specific genes previously identified as playing a role in AR was studied. Our results suggest significant modifications to the transcriptome in response to STS treatment that alter the expression of specific auxin-related genes and have a notable effect on the subsequent auxin application. As with previous reports, auxin transport activity emerges as a key factor in the induction of ARs in chestnut shoots.

## 2. Results

The first objective of this study was to investigate how modifications to ethylene signalling affected the rooting response of chestnut microshoots of mature origin (P2CR).

Table 1 shows how the application of STS in the phase prior to auxin treatment influenced root formation in chestnut shoots grown in vitro. Shoots were separated from the callus (wounding) and transferred to media without plant growth regulators with or without STS for 48 h. After this pre-treatment, shoots were treated with indole-3-butyric acid (IBA) for two additional days before being transferred again to hormone-free medium for rooting expression. Table 1 includes the response of controls without STS or auxin (T1), as well as shoots treated with STS and without auxin (T2), without STS and with auxin (T3), and with STS and auxin (T4).

**Figure 1:**
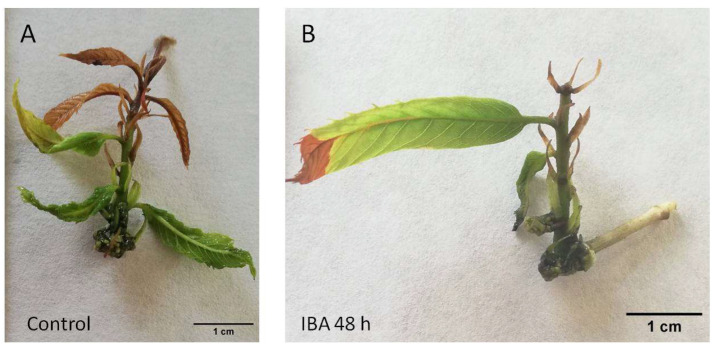
P2CR chestnut microshoots 30 days after the beginning of the treatments. (**A**) control microshoot with apical browning symptoms. (**B**) microshoot wounded (48 h) and then treated with IBA (25 µM) for another 48 h. IBA: indole-butyric acid.

**Figure 2:**
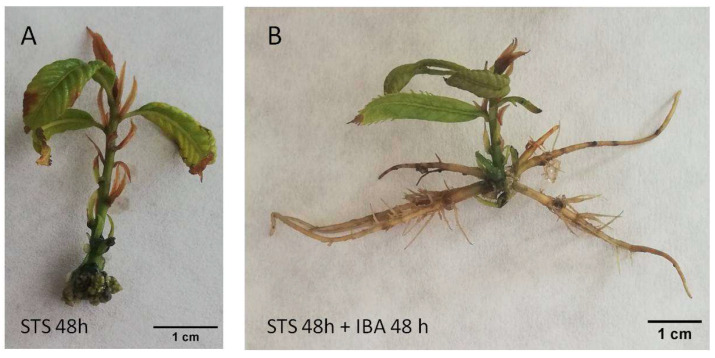
P2CR chestnut microshoots 30 days after the beginning of the treatments. (**A**) microshoot treated with STS (20 µM) for 48 h, showing an overall healthy aspect. (**B**) microshoot treated with STS for 48 h and then treated with IBA (25 µM) for another 48 h. STS: silver thiosulfate. IBA: indole-butyric acid.

Chestnut P2CR microshoots did not form roots when they were separated from the callus (wounded) and transferred to medium without plant growth regulators (control) or when they were wounded and only exposed to STS for 48 h without a subsequent auxin treatment (Table 1, Figure 1A and Figure 2A). Control microshoots show a poor health status and generally apical browning symptoms (Figure 1A). However, with STS application to the shoots, browning symptoms are reduced (Figure 2A), indicating that ET signalling inhibition improved the development of the shoots grown in the rooting media, although it failed to induce rooting in this material.

In the treatments in which auxin is applied after the pretreatments (T3, T4), the overall performance of the shoots was quite different. IBA alone produces a rooting percentage of 27%, with less than 2 roots per rooted shoot (Table 1). In addition, the shoots show asymmetric rooting systems that might not be adequate to support further growth of the plants (Figure 1B). When IBA was applied to microshoots pretreated with STS, the rooting rate was improved (54%), as well as the number of roots per shoot (Table 1). Similarly, the chestnut shoots developed a better root system architecture, with more roots that expanded in a symmetrical mode (Figure 2B). These results show a positive effect of STS pretreatment on the overall rooting performance of the chestnut shoots, especially in combination with auxin.

To uncover the molecular mechanisms accounting for the above physiological results, a transcriptomics experiment was conducted. Four treatments were selected: control and STS-treated samples (collected at 48 h), and IBA and STS + IBA samples (collected at 96 h). Long-read sequencing of the twelve libraries (four treatments, three replicates each) produced over 2.5 million sequences, with a mean read quality of 9 and no reads with quality below 7. The average read length was 597.7 bp. The reads were mapped against the *Quercus suber* genome v1.0, as in previous reports, retrieving a mean value of 66.61% of successfully mapped reads. After reading quantification, the differential expression analysis was developed in pairwise comparisons to look for specific differences among samples. With the objective of separating the effects of ET inhibition and auxin treatment, control shoots (collected after 48 h) were compared with STS samples (also collected at 48 h), whereas samples treated with IBA alone (96 h) were compared with STS + IBA samples (96 h).

**Figure 3:**
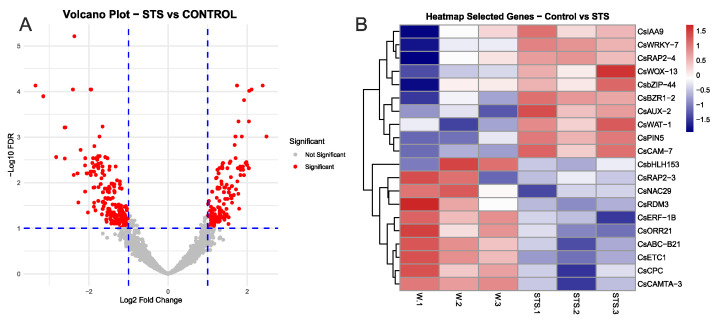
Main results from the comparison between chestnut shoot samples after 48 h of STS treatment. (**A**) volcano plot showing the groups of DEGs. (**B**) heatmap showing the normalized expression of selected genes from the groups of differentially expressed genes in the control and STS samples. Gene IDs for each gene are shown in Supplemental Table S3. FDR: false discovery rate.

When we contrasted control and STS samples, a total of 334 differentially expressed genes (DEGs) were identified, 200 upregulated in control samples and 134 in the STS samples (Supplementary Tables S1 and S2). A Volcano plot of this analysis is shown in Figure 3A, and the PCA analysis is shown in Supplementary Figure S1. From this group of DEGs, we selected specific genes relevant due to their putative role in the process, including transcription factors (TFs) and hormone-responsive genes, and developed a heatmap to show their expression. As shown in Figure 3B, genes responsive to different hormones were found among DEGs. In the case of the control samples, genes believed to be ET-responsive were found, including *CsRAP2-3* and *CsERF-1B*. In addition, a cytokinin-related transcriptional activator (*CsORR21*) and an auxin-linked transporter (*CsABC-B21*) were also found, as well as a relevant TF related to calmodulin (*CsCAMTA-3*) plus a gene linked to DNA methylation (*CsRDM3*). In STS samples, several genes linked with auxin transport (*CsPIN5*, *CsAUX-2*, *CsWAT-1*) and signalling (*CsIAA9*) were detected. Moreover, the brassinosteroid-responsive TF *CsBZR1-2* was also more expressed in these samples, as well as the *CsWOX-13* gene from the Wuschel family of TFs, involved in developmental processes. Overall, these results showed that the inhibition of ET signalling had a significant effect on the transcriptome of chestnut mature microshoots, particularly in genes related to auxin transport.

**Figure 4:**
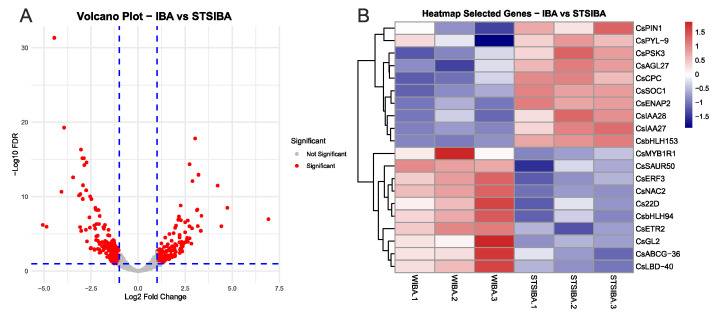
Main results from the comparison between IBA and STS + IBA samples. (**A**) volcano plot showing the groups of DEGs. (**B**) heatmap showing the normalized expression of selected genes from the groups of differentially expressed genes in the IBA and STS + IBA (STSIBA) samples. Gene IDs for each gene are shown in supplemental Table S6. FDR: false discovery rate.

The comparison between IBA and STS + IBA samples provided interesting results concerning the different effects of auxin treatment on the previously modified status of the microshoots. A total of 374 DEGs were identified here, which are shown in the Volcano Plot (Figure 4A), and the PCA plot is shown in Supplementary Figure S2. This analysis detected 195 DEGs upregulated in the IBA samples and 179 DEGs upregulated in the STS + IBA samples (Supplementary Tables S4 and S5). As in the previous analysis, selected genes were chosen from this group to show their expression (Figure 4B). In the IBA samples, ET-related genes were found, including a relevant TF (*CsERF3*) and a receptor (*CsETR2*). In addition, genes involved in auxin signalling (*CsSAUR50*, *Cs22D*) and in the transport of IBA (*CsABCG-36*) were identified, as well as other relevant TFs such as *CsLBD40* or *CsMYB1R1*. On the other hand, genes linked to auxin signalling from the Aux/IAA family (*CsIAA27*, *CsIAA28*) and the auxin efflux carrier *CsPIN1* were found in the group of DEGs of the STS + IBA samples. Other relevant genes here included two TFs from the MADS-box family (*CsAGL27*, *CsSOC1*) involved in developmental responses, a positive regulator of ET-responsive genes (CsENAP2), and the abscisic acid receptor *CsPYL9* (Figure 4B). Therefore, significant differences were detected in the effect of IBA treatment according to the pretreatments applied, with the results showing a complex interaction of phytohormones under both conditions.

**Figure 5:**
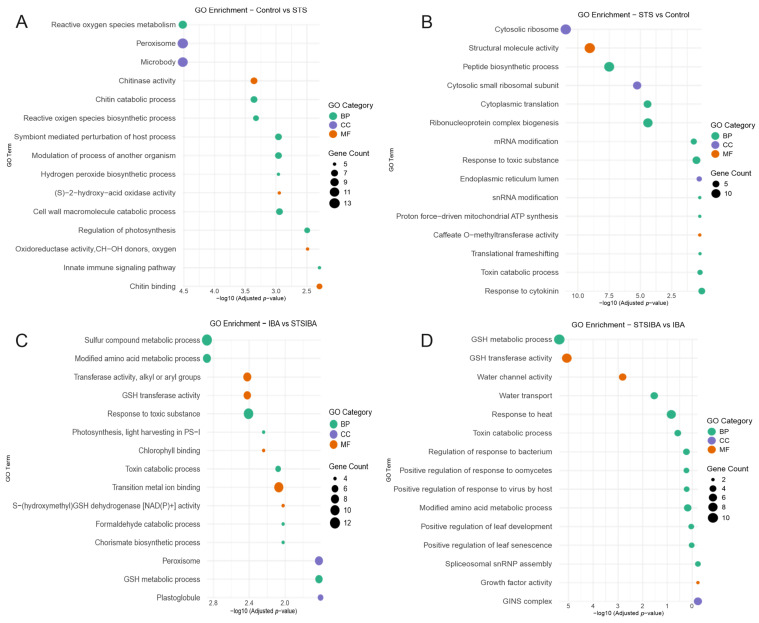
Gene Ontology (GO) analysis of the different groups of DEGs identified under the four different treatments. (**A**) main GO results from the DEGs identified in the control samples. (**B**) main GO results from the DEGs identified in the STS samples. (**C**) main GO results from the DEGs identified in the IBA samples. (**D**) Main GO results from the DEGs identified in the STS + IBA samples. IBA: indole-butyric acid. STS: silver thiosulfate. GSH: glutathione. BP: biological process. CC: cellular component. MF: molecular function.

To deepen our analysis of these groups of DEGs, a gene ontology study was developed for each of the four groups to identify the most relevant biological processes and molecular functions taking place under each condition. In the case of control samples, significant terms were mainly related to reactive oxygen species (“ROS metabolism”, “ROS biosynthetic process”, “Hydrogen peroxide biosynthetic process”, “(S)-2-hydroxy-acid oxidase activity”, “Oxidoreductase activity”), as well as linked to chitin (“Chitin metabolic process”, “Chitin binding”, “Chitinase activity”) biochemical processes and cell wall remodelling (Figure 5A). Meanwhile, in the STS samples, the majority of terms were linked to ribosomal activity and RNA synthesis (“Cytosolic ribosome”, “Ribonucleoprotein complex biogenesis”, “Peptide biosynthetic process”) and, interestingly, also “Response to cytokinin” (Figure 5B). Therefore, a 48 h STS treatment significantly modified the array of processes taking place at the base of the chestnut microshoots, with the control samples showing a high rate of ROS-related processes and exerting cell wall-modifying activities, while transcriptional processes were dominant in the STS samples.

In the IBA and STS + IBA samples, gene ontology enrichment analysis showed that under both conditions glutathione (GSH) related processes were relevant, with “GSH transferase activity” and “GSH metabolic process” common to both analyses, while in the IBA samples the molecular function “S-(hydroxymethyl)GSH dehydrogenase [NAD(P)+] activity” is also found (Figure 5C,D). In addition, photosynthesis-related terms were relevant in the IBA samples. In the STS + IBA samples, terms related to abiotic (heat, water) and biotic stress (bacteria, virus, oomycetes), as well as leaf development, could be identified. Therefore, gene ontology suggests that the auxin induction resulted in the activation of different signalling routes, whether there was a STS pre-treatment of chestnut mature microshoots or not, resulting in a significant difference in the rooting rate.

**Figure 6:**
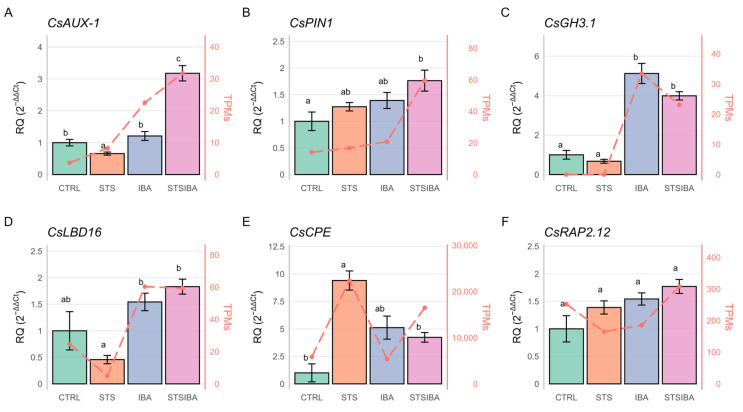
Quantitative real-time expression analysis of selected genes. (**A**) *CsAUX-1*. (**B**) *CsPIN1*. (**C**) *CsGH3.1*. (**D**) *CsLBD16*. (**E**) *CsCPE*. (**F**) *CsRAP2.12*. Different letters indicate statistical differences at *p* < 0.05. STS: silver thiosulfate. IBA: indole-butyric acid. TPMs: transcripts per million.

qRT-PCR analyses were performed on six genes to validate transcriptomics data (Figure 6). Among the selected genes, five had been previously found to be related to the AR process in chestnut (see Discussion). In addition, we also analyzed the expression of *CsAUX1*, an auxin influx carrier, due to the detection of a close homologue in the group of DEGs from the STS samples (Figure 3B). For this gene, a significant induction was detected in the STS + IBA samples, suggesting that auxin treatment effectively promoted gene expression, an effect not attained when ET signalling is not blocked (Figure 6A). Together with the expression data of the auxin efflux gene *CsPIN1* (Figure 6B), which also shows the highest expression in the STS + IBA samples, these findings suggest that the auxin transport machinery is more active after the auxin induction in STS-pretreated samples, allowing for the establishment of auxin gradients in mature tissues.

As expected, *CsGH3.1* was found to be induced by auxin, without significant differences in response to the STS treatment (Figure 6C). *CsCPE*, a histidine-rich protein previously found to be involved in morphogenetic responses, was notably induced by blocking ET signalling and also responsive to the auxin treatment according to its level of expression in the IBA samples (Figure 6E). *CsRAP2.12*, an ET-related TF, showed a non-significant increase in expression after STS and auxin treatments, suggesting a limited effect on the AR process in mature microshoots, at least at the time-points analyzed (Figure 6F). Finally, the *CsLBD16* TF form lateral organ boundaries domain family showed to be auxin-responsive, with STS treatment lowering its expression (Figure 6D). Overall, qRT-PCR results showed a good correlation with transcriptomic data and suggested that ET significantly affects auxin transport in chestnut mature microshoots.

## 3. Discussion

AR is a complex, multifactorial process in which different internal and external signals must be integrated for the successful development of a new organ. The light regime, the metabolic state of the tissues, Reactive Oxygen Species (ROS) signalling, and mineral nutrition all have a relevant impact on the outcome of the process. However, the activity of the different phytohormones is the most important factor in the formation of ARs. In the case of hard-to-root species, the need for an external auxin source, together with modifications to the hormone content derived from the wound applied to the tissues, establishes a new balance between these compounds that triggers specific gene expression and establishes a new developmental fate for specific cells. However, this new balance among phytohormones must occur at precise times and is also multifactorial in itself, as each hormone exerts a variable (although usually significant) level of influence on the homeostasis (i.e., Synthesis and transport) of the other phytohormones. Moreover, the interplay between hormones and stress signalling is key to defining cell fate [22].

The present study found that blocking ET signalling before auxin induction had a significant impact on the induction of ARs in mature, recalcitrant chestnut tissues. This is in agreement with previous results in which blockage of ET signalling or synthesis significantly enhanced the rooting response of P2CR mature chestnut shoots [21], although in that report the blockage treatment had been applied concomitantly with auxin instead of prior to IBA treatment, as in the present study. Interestingly, as with previous results, other rooting parameters such as root number and root length were also positively affected by ET signalling repression, showing a lasting effect on the performance of the shoots despite the time-limited duration of the treatment (Table 1; [21]). A positive effect of STS on the in vitro performance of several *Quercus* species has previously been reported [23], while in *Melia volkensii*, the application of STS with auxin improved rooting rates at some points, while it reduced callus formation and ameliorated shoot morphology [24], which is in agreement with our results. In Addition, in the P2CR clone, STS treatment for 48 h induced the expression of several auxin transport genes (*CsAUX-2*, *CsPIN5*) involved in polar transport between cells (Figure 3B), which might ease the effect of the subsequent induction with IBA. Therefore, ET seems to have an inhibitory activity on auxin transport genes, which might partly explain the limited effect of IBA induction. Nonetheless, other authors suggest that it is the activity of the liberated silver ions, rather than that of blocking ET, that is the main explanation for the effect of STS (or AgNO_3_) on auxin homeostasis. For instance, in Arabidopsis roots, silver ions seem to induce auxin efflux independently of ET signalling [25]. However, in our study, STS treatment had an effect on both auxin influx and efflux genes, or even on the vacuolar auxin transport gene *CsWAT1* (*Walls Are Thin-1*; [26]). Moreover, as shown for *CsAUX-1* expression, this auxin influx gene was also significantly induced after STS + IBA treatment (Figure 6A). Therefore, although the specific effect of silver ions on some transport genes cannot be ruled out, STS blocking of ET signalling seems to ease the expression of other components involved in auxin homeostasis.

Gene ontology analysis of the identified groups of DEGs provided some hints of the processes triggered by the different treatments. For instance, control samples show an enrichment in ROS signalling, chitin/chitinase pathways, and cell wall remodelling (Figure 5A), suggesting the existence of a wounding response linked with the formation of callus [27], which might reduce the ability of auxin to induce a rooting programme. On the other hand, ET signalling inhibition in the STS samples showed mainly an enrichment in transcriptional activity (Figure 5B), with no relevant ROS-related activity. ROS neutralization is considered relevant for cell cycle reactivation and, thus, regeneration [28]; therefore, the lack of ROS activity could aid in the promotion of the cellular competence for rooting. ET is known to play a relevant role in the repairing process [29], and an inhibition or lowering of this repairing-related activity might be positive for the induction of ARs. After IBA treatment, both IBA and STS + IBA samples show an enrichment in glutathione-related processes (Figure 5), a molecule known to be involved in root development and scavenging of ROS signalling [30]. However, STS + IBA samples also showed an increase in environmental stress responses, which are often linked to the formation of ARs [13]. Therefore, a lower level of ET signalling prevented the initiation of wounding-related responses that might lead to the formation of callus, while also elevating other stress signals putatively linked with an improvement in the AR response.

Other than auxin transport-related genes, the expression of genes previously linked with morphogenetic responses in chestnut was analyzed. *CsCPE*, a histidine-rich protein, had been related to somatic embryogenesis in *Quercus robur* [31]. Here, both STS and IBA treatments induced its expression, suggesting that it is also linked with morphogenetic responses in chestnut. *CsGH3.1* belongs to group II of the auxin-responsive GH3 gene family, involved in the formation of conjugates of auxin and amino acids, and whose members are related to several developmental processes [32]. As expected, the gene was auxin-responsive and apparently not under the control of ET. The levels of expression detected here are lower than those found in a previous report, probably because the IBA treatment used before applied a much higher concentration [33]. We also analyzed the expression of two TFs that have been proposed to play a role in the AR of chestnut shoots. On one hand, *CsRAP2.12* is a member of the Group VII of Ethylene Responsive Factors, which play a major role in responses under hypoxia conditions [34]. In previous reports, it was determined to be involved in AR of chestnut and oak microshoots, although to a greater extent in juvenile-like material at early time-points (6 h after the beginning of the treatment), and seemed to be more relevant in oak shoots [35]. Indeed, its expression was not significantly modified under the treatments applied here nor in a previous report using other ET-modifying compounds [21]. On the other hand, *CsLBD-16* belongs to the Lateral Organ Boundaries Domain family of TFs, which are related to different developmental processes. In particular, *LBD16* has been shown to be involved in callus and root formation [22], and recently it was shown to modulate pectin methylation during lateral root formation in Arabidopsis [36]. Previous reports on chestnut had shown that it is auxin-responsive and involved in AR in juvenile shoots, but initial induction might also be related to the formation of callus [19,37]. Results of the present study confirm this role of *CsLBD16* in regeneration responses, as it showed similar levels of induction in response to IBA irrespective of the pretreatment.

## 4. Materials and Methods

### 4.1. Plant Material and Culture Media

Plant material consisted of chestnut microshoots (P2CR) established from the crown branches of an 80-year-old chestnut tree and has been maintained in vitro for over thirty years [38] without relevant changes in multiplication traits (number of buds, growth rate, polyphenol content, etc.) [39]. While juvenile shoots from the same tree root successfully, the rooting of the P2CR shoots was maintained at very low rates (10%). Microshoots were multiplied in GD medium [40] supplemented with 0.1 mg/L of N^6^-benzyladenine (BA), 30 g/L sucrose, and 7 g/L Bacto Agar. The pH was adjusted to 5.7 prior to autoclaving at 120 °C for 20 min. The cultivation process was conducted in four-week cycles. After each cycle, well-developed microshoots were sub-cultured in fresh medium for a new multiplication cycle or used for rooting experiments. Cultures were incubated under a 16 h photoperiod with white fluorescent lamps providing a photosynthetic photon flux density of 50–60 µmol m^−2^ s^−1^ at 25 °C light/20 °C dark.

### 4.2. Rooting Experiments

The basal medium used for rooting experiments consisted of GD medium with one-third of the macronutrient concentration (GD^1/3^). The other components and characteristics were as specified above, except for the addition of BA, which is not present in rooting media. The in vitro plants were placed in jars with screw caps sealed with plastic film to prevent gas exchange. Silver thiosulfate (STS) 20 µM or indole-3-butyric acid (IBA) 25 µM were added to the rooting media depending on the treatments (Table 2). Before inoculation in the rooting medium, the calli of the microshoots were excised (wounding). Microshoots were incubated in darkness during the first 4 days of the experiment, and then transferred to fresh basal rooting media without plant growth regulators for 26 days in a 16 h photoperiod as described for multiplication.

For each treatment, three glass jars, with six microshoots per jar, and three replicates were used (*n* = 3 × 6 × 3 = 54 microshoots per treatment). After the 30-day rooting period, data were recorded for the percentage of rooted shoots, root number per explant, and the length of the longest root in each rooted shoot. Normality of the data was tested by the Shapiro–Wilk test, and the homoskedasticity was evaluated using the Levene test included in the “car” package [41]. The ANOVA test was used with Tukey’s HSD Post Hoc test when normality was confirmed. In the case of non-normal data, it was analyzed by the nonparametric Kruskal–Wallis test followed by Dunn’s test with Bonferroni adjustment from the “FSA” package [42]. When data is presented as a percentage, a regression analysis was developed assuming a beta distribution, instead of a normal one, using the “betareg” package [43], and the Post Hoc test was realized using the “emmeans” package [44]. All statistical analyses were performed in the R environment [45].

### 4.3. RNA Extraction, Transcriptomics, and Bioinformatics

Samples were collected at the indicated times and immediately frozen in liquid nitrogen and stored at −80 °C until further processing. Each sample consisted of at least 10 basal stem portions (approximately 1 cm). Control and STS-treated samples (STS) were collected after 48 h, while IBA and STS + IBA samples were collected 96 h after the beginning of the experiments (Table 2). Total RNA was extracted with the “FavorPrep Plant Total RNA Purification Mini Kit (for woody plants)” (Favorgen Biotech Corp., Pingtung, Taiwan). Following DNAase I treatment, the quality of the RNA was checked with a Nanodrop 2000c spectrophotometer and a Qubit 4 fluorometer (Thermo Fisher Scientific, Waltham, MA, USA). The transcript libraries were constructed with the PCR-cDNA Barcoding kit (SQK-PCB109, Oxford Nanopore Technologies—ONT) and then sequenced using a MinION device (ONT) with Flow Cells version R9.4.1. The software Guppy (v3.3.1) was used for basecalling the raw files and for demultiplexing. The adapters in the sequences were removed using Porechop v0.2.4 (https://github.com/rrwick/Porechop, accessed on 20 May 2025), and the quality was evaluated with FastQC (v0.12.1; [46]) and Nanoplot [47]. The resulting trimmed, high-quality reads were aligned and mapped using minimap2 (v2.24; [48]) against the *Quercus suber* reference genome (v1.0, [49]), with the option -ax splice. Quantification of the expression of the genes was developed with featureCounts (v2.0.5; [50]), and the differential gene expression analysis was completed with EdgeR (v4.2.1; [51]), setting the cutoff values at log2 Fold Change > 1 and the *p*-adjusted value at 0.1. The PCA analysis was performed by applying a log2 transformation of the read counts to stabilize variance. For gene ontology, protein sequences were analyzed with Pannzer [52], and results were further analyzed and visualized with ClusterProfiler (v4.12.6; [53]).

### 4.4. Quantitative Real-Time PCR

Quantitative Real-Time PCR (qRT-PCR) analyses were carried out to validate the expression of specific genes. The primers used for target and endogenous genes are designed using the Primer Designing Tool and Primer-BLAST software and can be found in Supplementary Table S7. Actin-2 (*CsACT-2*) and Elongation Factor 1 (*CsELF-1*) were used as reference genes. qRT-PCR analysis was performed with the same samples used in the transcriptomic experiments. 1 μg of total RNA per sample was reverse-transcribed, and then 1 μL of cDNA template (8.33 ng of input RNA) was amplified with the 2× Power SYBR^®^ Green PCR Master Mix (Applied Biosystems, Thermo Fisher Scientific, Waltham, MA, USA) in a final volume of 20 μL. The PCR thermal profile used was an initial step of 95° C/10 min, followed by 40 amplification cycles of 95° C/15 s, 60° C/1 min. Three biological and three technical replicates were assessed for each sample. Relative expression values were expressed as fold-change using the comparative CT method (ΔΔCT Method) [54].

## 5. Conclusions

In this report, we have shown that blocking of ET signalling significantly improves the rooting response of chestnut mature microshoots. STS treatment notably modified the transcriptome of the shoots, substantially changing the effect of the subsequent induction with auxin. Particularly, STS seemed to release the expression of several auxin transport-related genes from the inhibitory effect of ET, including both auxin influx and efflux genes. In Addition, it is worth noticing the lasting effect of STS treatment on the performance of the shoots, which also affected the development of the root system. Despite the significance of the results, given the rooting rate attained, it seems that other hormones in mature tissues prevent the auxin treatment from inducing a high-rooting response. Therefore, more research is needed to fully characterize the complex interactions of these compounds in chestnut, in order to achieve an optimized protocol for the vegetative propagation of this relevant species.

## Figures and Tables

**Table 1:** Effect of STS and auxin on the rooting response of P2CR chestnut microshoots.

Rooting Treatments (4 Days)			Rooting Performance (30 Days)		
Treatment	STS (0–48 h)	IBA (48–96 h)	Rooting (%)	Root No.	Root Length (cm)
T1. Control	0	0	0 c	0 c	0 b
T2. STS	20 µM	0	0 c	0 c	0 b
T3. IBA	0	25 µM	27.5 ± 2.75 a	1.25 ± 0.12 b	5.52 ± 0.41 a
T4. STS + IBA	20 µM	25 µM	54.3 ± 5.53 b	3.5 ± 0.14 a	7.32 ± 0.11 a

STS: silver thiosulfate. IBA: Indole-butyric acid. Values are presented as mean ± standard error, n = 54. For each parameter, different letters indicate significant statistical differences (ANOVA, *p* < 0.05).

**Table 2:** Experimental design and sequential media composition used during the induction and expression phases of adventitious rooting of P2CR microshoots.

	Rooting Induction Treatments		Rooting Expression
	(4 Days, Darkness)		(Up to 30 Days, Photoperiod)
	ET inhibition (0–48 h)	Auxin (48–96 h)	
T1. Control	GD ^1/3^	GD ^1/3^	GD ^1/3^
T2. STS	GD ^1/3^ STS 20 µM	GD ^1/3^	GD ^1/3^
T3. IBA	GD ^1/3^	GD ^1/3^ IBA 25 µM	GD ^1/3^
T4. STS + IBA	GD ^1/3^ STS 20 µM	GD ^1/3^ IBA 25 µM	GD ^1/3^

STS: silver thiosulfate. IBA: indole-3-butyric acid.

## Data Availability

The FastQ files generated for each of the samples have been submitted to the NCBI repository (https://www.ncbi.nlm.nih.gov/bioproject, accessed on 1 December 2025) under accession ID PRJNA1083332.

## Supplementary Materials

The following supporting information can be downloaded at: https://www.mdpi.com/article/10.3390/plants14243756/s1, Figure S1: PCA analysis of Control vs. STS samples; Figure S2: PCA analysis of IBA vs. STSIBA samples; Table S1: Differentially Expressed Genes in the Control samples; Table S2: Differentially Expressed Genes in the STS samples; Table S3: List of genes in Figure 3B; Table S4: List of Differentially Expressed Genes in the IBA samples; Table S5: Differentially Expressed Genes in the STS + IBA samples; Table S6: List of genes in Figure 4B; Table S7: Primers used for qRT-PCR expression analysis.
