# Supplementary figures and images for: Pretreatment with Silver Thiosulfate Increases the Auxin-Inductive Effect for Rooting Mature Chestnut Shoots
Source: Plants (Basel). 2025 Dec 10;14(24):3756. doi: 10.3390/plants14243756 (PMC12736431; doi:10.3390/plants14243756)

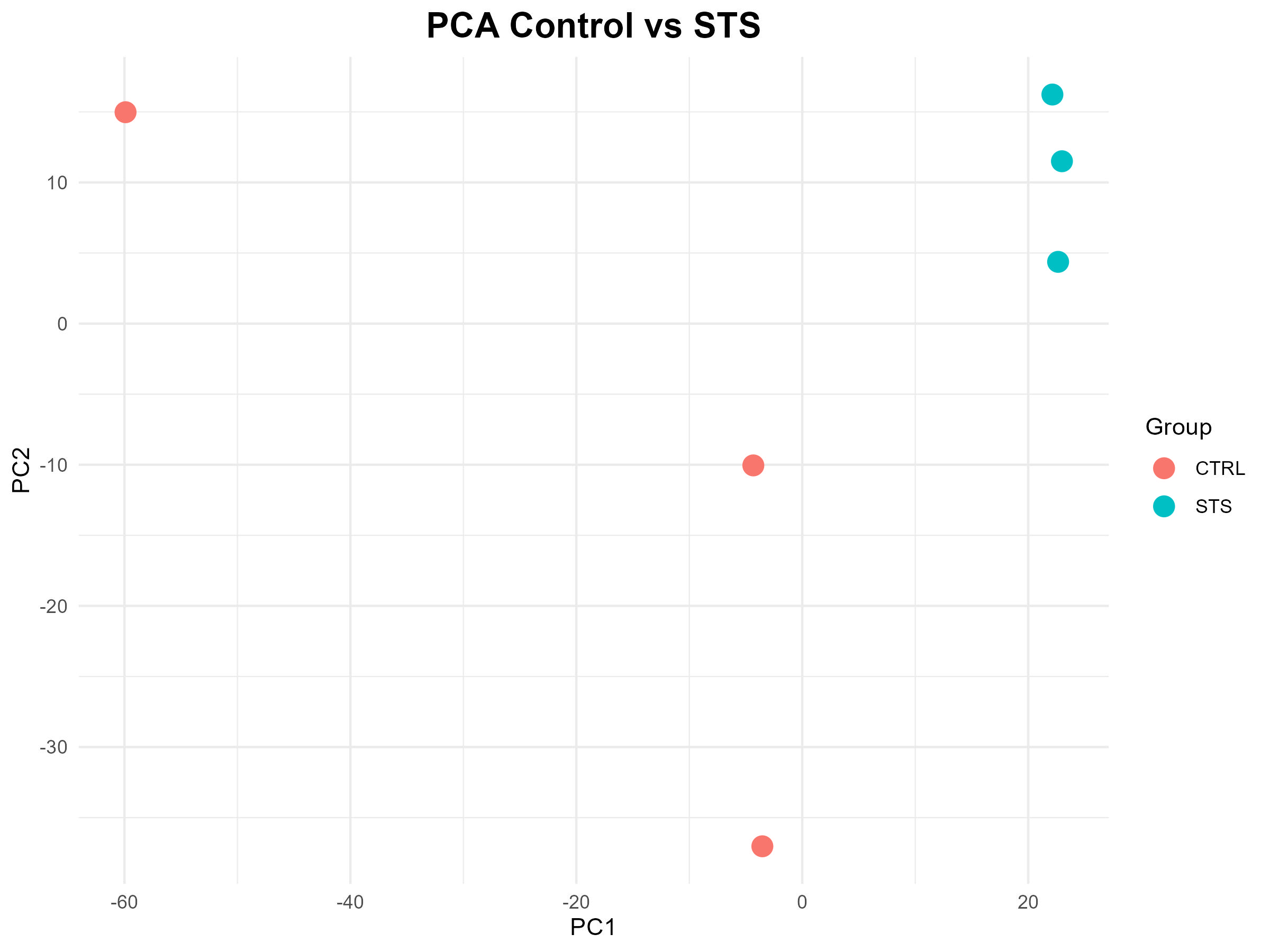

Supplement: Supplementary file 1 [file plants-14-03756-s001.zip › Supp. Figure S1 - PCA Control vs STS.jpeg]

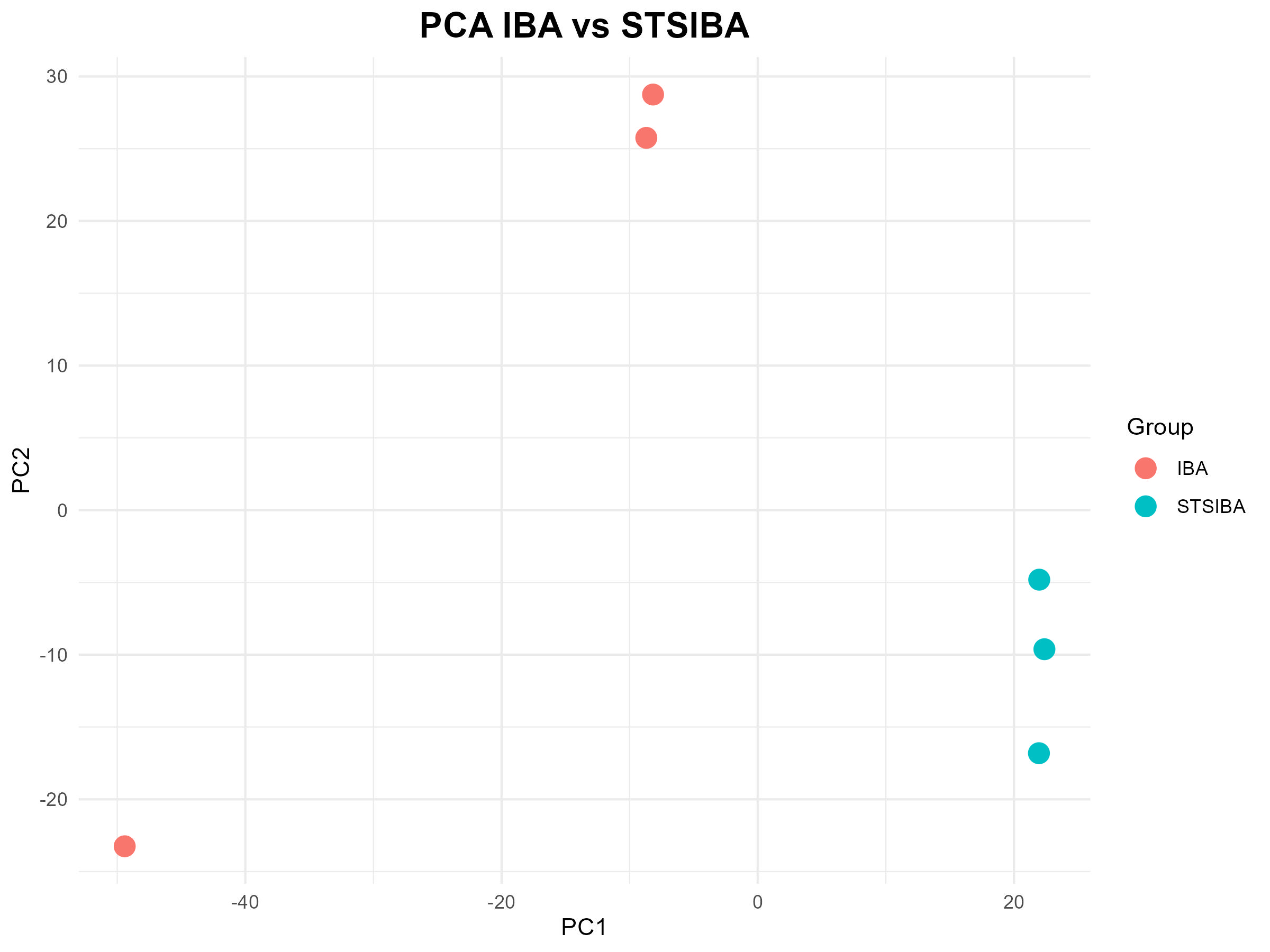

Supplement: Supplementary file 1 [file plants-14-03756-s001.zip › Supp. Figure S2 - PCA IBA vs STSIBA.jpeg]
